# An economic analysis of human milk supplementation for very low birth weight babies in the USA

**DOI:** 10.1186/s12887-019-1691-4

**Published:** 2019-09-14

**Authors:** Grace Hampson, Sarah Louise Elin Roberts, Alan Lucas, David Parkin

**Affiliations:** 1 Office of Health Economics, 7th Floor, Southside, 105 Victoria St, London, SW1E 6QT UK; 2 King’s College London, London, UK; 3 Institute of Child Health, University College London, London, UK; 4 City University of London and Office of Health Economics, London, UK

**Keywords:** Exclusive human milk diet, Cost-effectiveness, Nutrition, Infants, Preterm, Economics

## Abstract

**Background:**

An exclusive human milk diet (EHMD) using human milk based products (pre-term formula and fortifiers) has been shown to lead to significant clinical benefits for very low birth weight (VLBW) babies (below 1250 g). This is expensive relative to diets that include cow’s milk based products, but preliminary economic analyses have shown that the costs are more than offset by a reduction in the cost of neonatal care. However, these economic analyses have not completely assessed the economic implications of EHMD feeding, as they have not considered the range of outcomes affected by it.

**Methods:**

We conducted an economic analysis of EHMD compared to usual practice of care amongst VLBW babies in the US, which is to include cow's milk based products when required. Costs were evaluated from the perspective of the health care payer, with societal costs considered in sensitivity analyses.

**Results:**

An EHMD substantially reduces mortality and improves other health outcomes, as well as generating substantial cost savings of $16,309 per infant by reducing adverse clinical events. Cost savings increase to $117,239 per infant when wider societal costs are included.

**Conclusions:**

An EHMD is dominant in cost-effectiveness terms, that is it is both cost-saving and clinically beneficial, for VLBW babies in a US-based setting.

## Background

Very low birth weight (VLBW) babies, particularly those with a birth weight below 1250 g, are at risk of major clinical complications, including necrotising enterocolitis (NEC) and systemic sepsis. NEC is a leading cause of death for these babies [1], and has long term health consequences amongst those who survive, often leading to impaired neurodevelopment.

VLBW babies have substantially greater nutrient requirements than full term babies to fuel their growth, including the growth of the preterm brain. Maternal milk, expressed and fed by nasogastric tube, is recommended for them because breast milk has good enteral feed tolerance and favourable effects on clinical course and later outcomes. However, human milk alone does not meet the nutritional needs of these babies. It requires fortification with a specially designed fortifier, and if the mother does not express sufficient breast milk to meet volume requirements, a nutrient enriched “preterm formula” is required in addition.

Milk fortifiers and preterm formulas routinely used in preterm infant feeding are derived from cow’s milk, which is associated with adverse outcomes in these babies – notably poorer feed tolerance by the immature gut, sepsis, NEC, bronchopulmonary dysplasia (BPD), retinopathy of prematurity and neurodevelopmental problems. This is the usual practice of care in at least 50% of the neonatal intensive care units (NICUs) in the US [2].

However, there are alternatives manufactured entirely from donor human milk. The clinical value of an exclusive human milk diet (EHMD), using mother’s milk supplemented where necessary by fortifiers and formulas manufactured from donor human milk, has been supported by two key clinical trials [3–5]. These demonstrated that an EHMD results in substantially improved outcomes, with reductions in NEC, sepsis and bronchopulmonary dysplasia (BPD).

As Johnson et al. [6] state, VLBW babies are very expensive to treat, so there is a potential for large cost reductions if time in the NICU could be reduced and other costly interventions avoided. Clinical data suggest that many expensive adverse clinical outcomes could be prevented by using an EHMD, which would not only greatly improve the outcome of these vulnerable infants but also potentially lower treatment costs. However, human milk-based products are more expensive to purchase than those derived from cow’s milk, so it is important to consider the extent to which this is offset by – or perhaps is outweighed by – any reduction in treatment costs.

Published economic analyses of EHMDs have analysed the impact on net health care costs, but these are restricted to costs and outcomes in the first few years of life, and are generally limited to treatment costs related to NEC and sepsis [7–10]. This paper aims to provide a more complete economic evaluation of the impact of an EHMD in the US, including both the immediate costs of treatment, a broader range of subsequent clinical events (NEC, late onset sepsis, short bowel syndrome, BPD, retinopathy of prematurity) and longer term costs of retinopathy of prematurity and neurodevelopmental problems, specifically cerebral palsy.

## Methods

### Model overview

An economic model was undertaken to explore the costs and benefits of using an EHMD in VLBW babies, compared to usual practice of care in which cow’s milk based products are used. The model was based on a variant of economic evaluation called cost-consequence analysis, in which estimates of cost-effectiveness are supplemented by further information on wider costs and benefits so that decision-makers may form their own judgements on which feeding strategy offers greatest value in terms of costs and benefits. The main analysis, or ‘base case’, considers the clinical effects of an EHMD, as well as the costs to the health care payer of the diet (including the costs of the initial hospital stay and follow up from treatment of retinopathy of prematurity and cerebral palsy), and the subsequent clinical effects. Sensitivity analyses explore the additional costs to society of cerebral palsy and retinopathy of prematurity. Future costs and benefits are discounted to their present values in 2016 using an annual discount rate of 3%, as recommended by the US Second Panel on Cost-Effectiveness in Health and Medicine [11].

The model uses a hypothetical population of 1000 VLBW babies, all of whom are assumed to be admitted to a NICU. They are assigned to either an EHMD or to usual practice of care. The babies may then develop NEC, late onset sepsis, both of these, or neither, with the probability of each event dependent on the diet that they have received. Babies who develop NEC can be treated medically or surgically.

All babies also have a probability of developing the following sequelae: BPD, a chronic lung disease; retinopathy of prematurity, which causes abnormal blood vessels to grow in the retina and can lead to blindness; and cerebral palsy, a neurodevelopmental problem. Babies who receive surgical NEC treatment can also develop short bowel syndrome, a condition which can lead to long term or lifelong parenteral feeding. The probability of developing these adverse outcomes is assumed to differ according to whether the baby received usual practice of care or an EHMD.

**Fig. 1:**
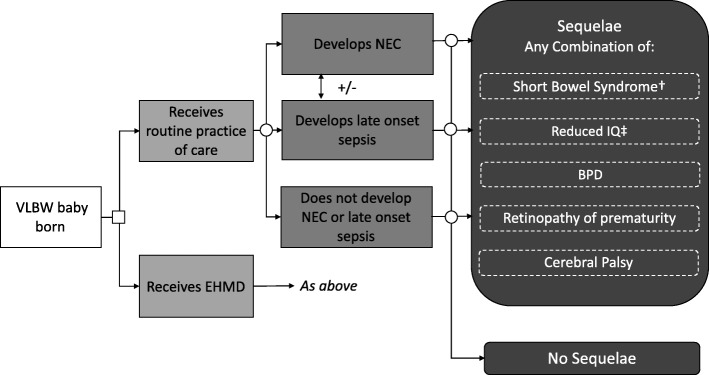
Diagram of model. Notes: Babies can also die during the initial hospital stay. For simplicity, this is not shown in the diagram; †short bowel syndrome is only a possibility for babies who have undergone surgical necrotising enterocolitis treatment; ‡reduction in IQ is applied to babies with necrotising enterocolitis and/or late onset sepsis only (costs to the health care system are limited, thus costs are only included in sensitivity analysis where we consider the societal perspective)

Figure 1 shows the structure of the model. The model takes the form of a decision tree and uses a cohort approach. It was developed in Microsoft Excel©.

### Diets

Babies in the model receive one of the following diets:

1. Usual practice of care: babies are fed with mother’s expressed breast milk supplemented with a cow’s milk based fortifier. When a mother expresses insufficient milk to meet her baby’s needs a cow’s milk based preterm formula is used, although if donor human milk is available, this may be used exclusively or as part of overall feeding.
2. EHMD: babies receive an EHMD. Babies are fed with mother’s expressed breast milk supplemented with a human milk based fortifier. When a mother expresses insufficient milk to meet her baby’s needs, and donor human milk is available, this is fortified with a human milk based fortifier. When donor milk is not available a human milk based preterm formula is used.

All other aspects of care are assumed to be the same between groups.

### Clinical inputs

The model is populated using the results of a literature search undertaken to identify parameters for the model. See Table 1 for full details.

**Table 1:** Key clinical parameters

Description	Base case [reference]	Sensitivity analysis	
		Lower cost scenario	Higher cost scenario
Probability of event in the usual practice of care group (%)			
Necrotising Enterocolitis Of which surgically treated	16.7 [12]	17.2 [5]	16.7 [12]
	63.5 [12]	68.8 [5]	63.5 [12]
Late onset sepsis	30.3 [12]	34.4 [5]	30.3 [12]
Mortality (during initial hospital stay)	17.2 [12]	7.5 [5]	17.2 [12]
Bronchopulmonary dysplasia	56.3 [12]	30.1 [5]	56.3 [12]
Retinopathy of prematurity	9.0 [12]	10.7 [13]	9.0 [12]
Relative risk of event in EHMD group			
Necrotising Enterocolitis	0.31 [5]	0.31 [5]	0.41 [12]
Late onset sepsis	0.87 ^a^ [5]	0.63 [12]	0.87 ^a^ [5]
Mortality (during initial hospital stay)	0.79 [12]	0.79 [12]	0.24 [5]
Bronchopulmonary dysplasia	0.99 [5]	0.85 [12]	0.99 [5]
Retinopathy of prematurity	0.15 [13]	0.15 [13]	0.58 [12]
Probability of event independent of treatment group (%)			
Short bowel syndrome (following surgical NEC only)	15.7 [14]	–	–
Cerebral palsy^b^			
No NEC or late onset sepsis	14.8^c^ [15]	–	–
Following late onset sepsis	14.8^c^ [15]	–	–
Following NEC (odds ratio)	1.55 [15]	–	–
Reduction in IQ points following:			
NEC	11 [16]	–	–
Late onset Sepsis	9 [17]	–	–

Abbreviations: *EHMD* Exclusive Human Milk Diet, *NEC* Necrotising Enterocolitis

Notes: ^a^data for sepsis rather than late onset sepsis specifically; ^b^When an infant has both NEC and late onset sepsis the higher of the two probabilities of developing CP is used; ^c^Control group included late onset sepsis patients

The ‘Favourable to EHMD’ column includes the data (RCT or cohort) under which EHMD would have the greatest relative benefits and cost savings. The favourable and least favourable data are included in sensitivity analyses

The literature review was based on bidirectional citation searching [18] starting from two key papers in this area [5, 12] that were known to the authors, and supplemented by searches in Google Scholar. We looked for RCTs or observational studies that compared clinical outcomes following an EHMD compared to standard of care. We also considered studies that provided evidence of clinical outcomes resulting from NEC or sepsis, as these are key clinical outcomes for EHMD.

The two key studies were:

1. a combined analysis [5] (*n* = 260) of patient level data from the two (aforementioned) key randomised controlled trials (RCTs) of an EHMD compared to usual practice of care (as defined above) in VLBW babies [3, 4].
2. a large (*n* = 1587) retrospective cohort study of EHMD compared to usual practice of care [12] across four centres in the US, in which data were collected before and after the introduction of an EHMD.

A third RCT [13] has also included the retinopathy of prematurity outcome, which was not included in these two RCTs. No additional RCTs were found via the literature search.

Best practice in economic modelling is to use clinical practice data to estimate clinical outcomes in the comparator group - in this case, those receiving usual practice of care - and to apply relative treatment effects from RCTs to estimate outcomes in the intervention group – in this case, those receiving an EHMD. The retrospective cohort study [12] provides the best clinical practice data available to us to represent infants receiving usual practice of care as defined above. Whilst other data is available [14, 15] on the incidence of NEC in this population, it does not differentiate between babies receiving bovine-based fortifier and an EHMD. The preventative effect of an EHMD on NEC, mortality and RoP incidence was stronger in the RCTs than in the retrospective study, whereas the relative effect of EHMD on BPD and late onset sepsis was stronger in the retrospective study, although note that the RCTs report sepsis rather than late onset sepsis. We use the RCT data on relative effects in the base case, and utilise alternative values in a sensitivity analysis. There is one exception to this, where for mortality we use estimates from the retrospective cohort study [12] to estimate mortality in the usual practice of care and EHMD groups in the base case. This is because, for this outcome, combining the data sources resulted in what appeared to be an unrealistically high number of lives saved by the EHMD. These results are presented separately as a sensitivity analysis.

The three key papers did not provide data on the probability of developing cerebral palsy or short bowel syndrome, or the impact on survivors’ subsequent IQ. The probability of developing these outcomes were thus based on the best available evidence identified through the literature search.

There is currently insufficient evidence about the direct impact of an EHMD on the probability of developing cerebral palsy. In our model, this outcome therefore depends only on whether the baby developed NEC, late onset sepsis or both during the initial hospital stay. The data are obtained from a meta-analysis of 4377 VLBW babies with and without NEC and sepsis across five cohort and case-control studies [15].

To calculate the incidence of short bowel syndrome following surgical NEC, we used data from Cole et al. (2008) [19], who conducted an analysis of 12,316 VLBW babies. They found that 0.7% of the cohort had short bowel syndrome, 96% of which was caused by NEC. We assume that all these NEC-related cases of short bowel syndrome are due to surgical NEC, and calculated the incidence rate of short bowel syndrome amongst babies with surgical NEC accordingly. Some of these patients would require lifelong Total Parenteral Nutrition or an intestinal transplant, but we were unable to include these long term effects in the model because of a lack of robust data on their incidence.

The reduction in IQ points resulting from NEC and late onset sepsis was taken from Roze et al. [16] and Van der Ree et al. [17] respectively. Babies who had both NEC and late onset sepsis were assumed to experience the loss of IQ associated with NEC only, as this was the higher of the two estimates. We do not assume any additive effect.

In each case, the assumptions are conservative about the impact of an EHMD, in that they are likely to underestimate its beneficial effects, although the number of patients likely to be affected will be small.

### Costs

The model includes the costs of the diets, plus the costs of treating any complications that arise during the initial hospital stay. We also include costs for follow up from treatment of retinopathy of prematurity, and lifetime costs to the health care payer of cerebral palsy. Using this approach, the long-term health care costs following the initial hospital stay and resolution of NEC, late onset sepsis and sequelae for all patients in the model who do not have cerebral palsy or retinopathy or prematurity is assumed to be the same for all babies. Table 2 shows the key cost parameters used in the model. As before the costs were identified via bidirectional citation searching and Google scholar, starting from two key papers that were known to the authors [6, 7], with the aim of identifying the most recent and applicable cost estimates. The cost of the EHMD was provided by Prolacta Bioscience. All costs were expressed in 2016 USD prices, updated using the medical care component of the Consumer Price Index [24].

**Table 2:** Key cost and resource use parameters

Description	Base case parameter value [reference]
Quantities of milk and formula (median)	
EHMD:	
Mother’s milk	1943 mL [5]
Donor milk	883 mL [5]
Usual practice of care:	
Mother’s milk	2102 mL [5]
Formula	2109 mL [5]
Cost of diet	
Prolact+ 6® (30 mL)	$187.50^a^
Donor milk (1 l)	$183 [20]
Total cost of diet	
EHMD	$7731
CMD	$226 [21]
Cost of initial stay in hospital for VLBW baby	
No NEC, late onset sepsis or sequelae	$49,660 [6]
Incremental costs for NEC, late onset sepsis and sequelae	
NEC (surgically treated)	$229,431 [21]
NEC (medically treated)	$85,734 [21]
Late onset sepsis	$12,413 [6]
Bronchopulmonary dysplasia	$38,966 [6]
Retinopathy of prematurity	$5939 [22]
Short bowel syndrome	Included in surgical NEC costs
Cerebral palsy	$147,268 [23]

Notes: ^a^Source: communication from Prolacta Bioscience

Abbrevaitions *EHMD* Exclusive Human Milk Diet, *NEC* Necrotising Enterocolitis

Different strengths of human milk fortifier are available. The exact product used is likely to differ according to local protocols, and in many cases will change throughout the infant’s stay. We made the simplifying assumption that the Prolact+ 6® is used; this product is mid-range in terms of strength and cost and is the most commonly used product [Prolacta, personal communication]. The + 6 is 30 mL and is mixed with 70 mL of donor milk. The cost of 30 mL Prolact+ 6 product in the US is $187.50, and the cost of donor milk was assumed to be $133 per litre in 2008$ (updated here to $183 in 2016$), based on a retrospective evaluation of the use of donor milk for feeding very preterm infants in the NICU [20]. The estimated total cost of the EHMD is therefore $7731.

The cost of cow’s milk based products as reported in Ganapathy et al. [21] ($195 in 2011$, updated here to $226 in 2016$) was subtracted from the cost of the EHMD to give an estimate of the incremental cost of the EHMD.

The incremental costs of NEC, late onset sepsis, BPD and retinopathy of prematurity were taken from economic analyses of treatments of these conditions in the US [6, 21, 22]. The cost of short bowel syndrome was included in the cost of surgically treated NEC. These costs were added to the cost of an NICU stay for a VLBW baby with no complications, provided by a retrospective analysis of the costs of comorbidities amongst 425 VLBW babies [6]. Costs were obtained after controlling for birth weight, gestational age, sociodemographic characteristics, and various clinical outcomes.

The discounted lifetime costs of cerebral palsy were calculated from data provided by the Centres for Disease Control and Prevention [23] to the health system.

Note that all costs are incremental to the cost of a baby that does not develop NEC, late onset sepsis or sequelae. The costs are considered to be additive in all cases: data from Johnson et al. [6] suggests that this is a reasonable simplifying assumption as treatment costs significantly increase with the number of morbidities.

### Sensitivity analysis

Sensitivity analyses are undertaken to explore the impact on the results if alternative input values are used in the model, for example different baseline estimates of NEC prevalence. We conducted four types of sensitivity analysis:

1. We conducted various threshold analyses to explore the incidence rates of late onset sepsis and NEC that would be required for the EHMD to be cost-saving.
2. We present lower and higher cost scenarios. The lower cost scenario uses the data (RCT [5, 13] or cohort [12]) under which EHMD would give the greatest cost savings, and the higher cost scenario those data which would produce the least savings. The parameters used for these scenarios are provided in Table 1. Because of the conservative modelling approach that we adopted, the higher cost scenario is very similar to the base case.
3. We included some examples of wider societal costs for which data was available. These are the societal costs of cerebral palsy [22], including costs of health care, specialised child care, specialised education, housing and lost productivity and reductions in lifetime earnings [25] which can be attributed to lower IQ resulting from NEC and sepsis [16, 17] and to retinopathy of prematurity via productivity losses of caregivers and blind people [21]. This analysis does not capture the full societal benefits of using an EHMD, as societal savings from reductions in NEC, late onset sepsis and long term consequences of short bowel syndrome are not included due to data limitations. We also only consider lost productivity amongst survivors, and not amongst non-survivors.
4. As mentioned previously, we present the case where the mortality for the usual practice of care group is estimated from the retrospective cohort study [12], with the treatment effect of an EHMD on mortality taken from trial data [5].

## Results

### Clinical and cost-effectiveness

Tables 3 and 4 show the results for the base case analysis. An EHMD reduces occurrence of most adverse clinical outcomes during the initial hospital stay, including preventing 36 deaths in the hypothetical 1000 infant cohort, but not BPD. The increased incidence of BPD is because more babies survive with a EHMD. This reduction in mortality outweighs the reduction in risk of getting BPD.

**Table 3:** Clinical results

Event	Incremental number of events per 1000 babies (EHMD – usual practice of care)
Deaths (initial hospital stay)	− 36
Cases of NEC	− 115
Medical	−66
Surgical	−94
Cases of late onset sepsis	−39
Cases of bronchopulmonary dysplasia	18
Cases of retinopathy of prematurity	−63
Cases of Cerebral palsy	−2
Cases of Short bowel syndrome	−15

Abbreviations: *EHMD* Exclusive Human Milk Diet, *NEC* Necrotising Enterocolitis

**Table 4:** Cost results

Costs (per person)	EHMD	Usual practice of care	Incremental (EHMD – usual practice of care)
US analysis			
Diet	$7731	$226	$7505
Baseline hospital costs	$49,660	$49,660	$0
NEC and late onset sepsis	$9479	$33,310	-$23,832
Sequelae	$38,261	$38,242	$18
Total	$105,130	$121,438	-$16,309

Abbreviations: *EHMD* Exclusive Human Milk Diet, *NEC* Necrotising Enterocolitis

In addition to clinical improvements, the EHMD generates overall lower health care costs, because the lower costs due to the reduction in adverse clinical outcomes more than offset the increase in dietary costs. This means that the EHMD is dominant in cost-effectiveness terms in a US setting.

### Sensitivity analysis

**Fig. 2:**
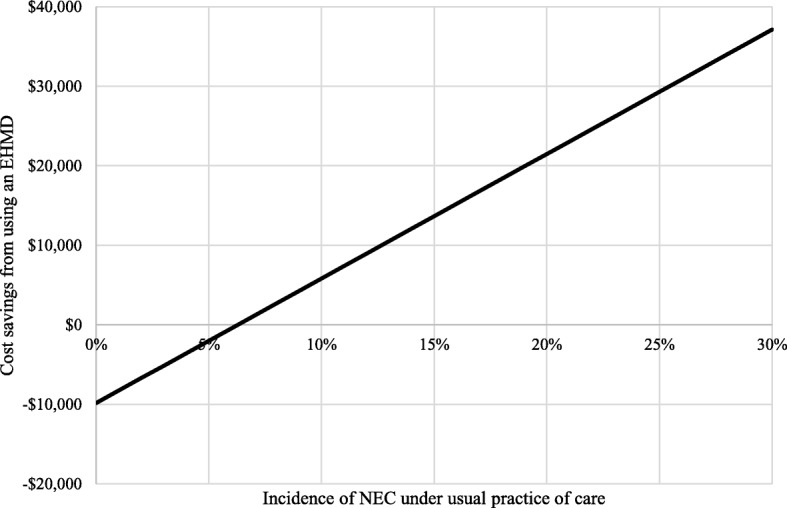
Incremental cost savings (per infant) from using an EHMD compared to usual practice of care, according to the incidence of NEC under usual practice of care

Holding other factors constant, an EHMD would still reduce costs if the baseline incidence of NEC in the usual practice of care group was as low as 7%. This is shown diagrammatically in Fig. 2. Below a baseline incidence of 7% the EHMD leads to small increases in cost per patient ($2010 at 5%; $6707 at 2%) but still gives significant clinical benefits, for example at 5% 34 cases of NEC would be avoided, at 2%, 14 cases. Ignoring all other clinical benefits, this means $59 per case prevented at 5% incidence and $479 at 2%. The EHMD would still be cost saving if the baseline incidence of late onset sepsis was zero. Any increases in the incidence of NEC or late onset sepsis increase the cost savings associated with EHMD.

**Fig. 3:**
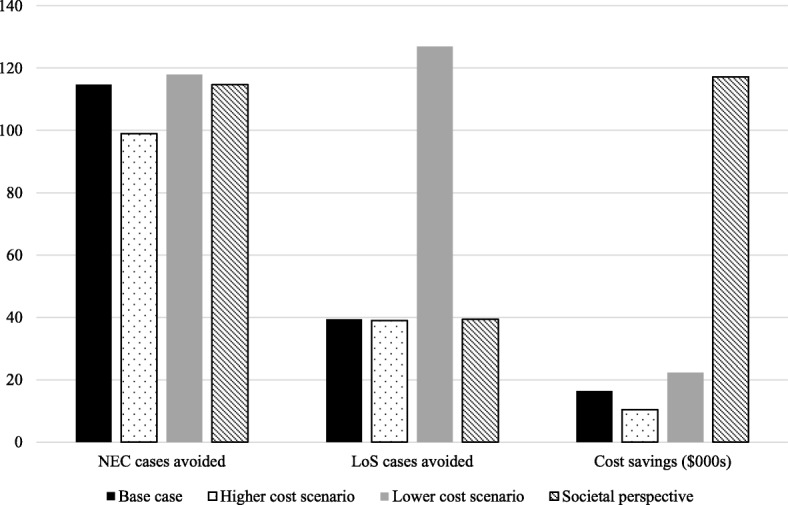
Results of sensitivity analyses

When wider societal costs are included the cost savings from an EHMD are even greater at $117,239 per infant. In the case lower cost scenario, the cost savings per infant increase to $22,226, and in the higher cost scenario they reduce to $10,416 (Fig. 3). Finally, when the retrospective cohort data and trial data are combined to estimate mortality for the EHDM group, an additional 131 lives are saved per 1000 babies in the EHMD group, leading to a decrease in cost savings ($12,164 per infant).

## Discussion

Our analysis suggests that the use of an EHMD for VLBW babies dominates in cost-effectiveness terms in a US setting. The clinical benefits calculated by the model are substantial, and in line with expectations based on the input data and published literature in this area [3–6]. The finding of dominance holds in both the higher and lower cost scenarios which are based on alternative data from the literature. Cost savings increase when wider societal costs are included, and remain positive as long as the baselines incidence of NEC is 7% or above. The analysis is based on conservative assumptions and therefore provides a lower limit to the estimate of cost-effectiveness of an EHMD. For example, we have not included the long-term costs or health care consequences of short bowel syndrome, including the possibility of requiring a transplant. This event is more common following usual practice of care than EHMD (see Table 1) and can both be very expensive and have a catastrophic impact on a survivor’s quality of life. We have also not included in our analysis all possible long term outcomes, such as reduced risk of cardiovascular disease in the EHMD group. This means that the true cost savings and health improvements of an EHMD are likely to be even greater than those estimated here.

The model demonstrates that use of a EHMD has a substantial impact on mortality, a reduction of 36 deaths in 1000 babies. This is directly in line with the two key papers that provide clinical data on this topic [5, 12]. In addition, the relative risk implied by our data and results is 0.79, which is within the 0.06-1.16 range identified by Bhutter et al. [26] in a review of different interventions to prevent neonatal mortality.

The model also shows that use of a EHMD could increase the number cases of BPD by 18 in every 1000 babies, despite the fact that there is a greater probability that babies given usual practice of care will have BPD compared to those given an EHMD. The reason is that this is outweighed by the lower mortality for those given an EHMD, increasing the population at risk of developing BPD.

When a health care technology or intervention has an impact on both mortality and morbidity of different kinds, a valuable composite measure of outcomes is the change in patients’ Quality Adjusted Life Years (QALYs) [11]. Because many of the outcomes of an EHMD may be long-term, this would require us to calculate QALYs over each baby’s lifetime. The necessary data on lifetime quality of life and mortality rates do not exist, and would be difficult to collect, as mortality rates are often only recorded during the NICU period, quality of life is not easily elicited for neonates and young children and collecting subsequent quality of life data would require long-term follow up studies. We could therefore not calculate QALY changes, but note for future research that this would be a valuable addition to current outcome measures.

The analysis was further constrained by the availability of relevant data on all variables relevant to a cost-and-consequences analysis. For example, we could not identify any data on the direct impact of the EHMD on the probability of developing cerebral palsy, and thus had to model this through the impact of NEC and late onset sepsis.

In terms of model validation, we considered the Assessment of the Validation Status of Health-Economic decision models (AdViSHE) tool [27]. The tool does not give a validation score, but invites model developers to think through various elements of validation of the conceptual model, data inputs, the computerized model, and operational aspects. The model has undergone cross validity testing with other conceptual models; extreme values testing and tracking of patients through the model; and validation in comparison to alternative analyses and using alternative input data. Validation could be further built upon by seeking additional validation on the choice of input variables and results with a panel of clinical experts. This was not within scope of the current analysis.

We cannot draw any strong conclusions on the generalisability of these results to other settings, as the clinical and resource use data are all specific to the US. The extent of the cost-savings shown by our analysis suggests that it is worth investigating the likelihood that an EHMD is cost-effective in other settings. We are aware that RCTs are underway in Europe which will provide useful information on the impact of an EHMD in a public health care system.

## Conclusion

This analysis demonstrates that an EHMD is dominant in cost-effectiveness terms, that is it is both cost-saving and clinically beneficial, for VLBW babies in a US-based setting. These findings indicate that use of an EHMD rather than usual practice of care in a US setting would reduce costs for the health care payer and lead to improved health outcomes for VLBW babies.

## Data Availability

Data sharing is not applicable to this article as no datasets were generated or analysed during the current study.

## Abbreviations

BPD: Bronchopulmonary dysplasia
EHMD: Exclusive human milk diet
NEC: Necrotising enterocolitis
NICU: Neonatal intensive care unit
QALYs: Quality Adjusted Life Years
RCT: Randomised controlled trial
VLBW: Very low birth weight
